# Revitalizing Rural Health Care Delivery: Can Rural Health Practitioners be the Answer?

**DOI:** 10.4103/0970-0218.45368

**Published:** 2009-01

**Authors:** Kapil Yadav, Prashant Jarhyan, Vivek Gupta, Chandrakant S Pandav

**Affiliations:** Centre for Community Medicine, Old OT Block, All India Institute of Medical Sciences, New Delhi, India

The rural health system of India is plagued by serious resource shortfall and underdevelopment of infrastructure leading to deficient health care for a majority of India. The differences in urban-rural health indicators are a harsh reality even today; infant mortality rate is 62 per thousand live births for rural areas as compared to 39 per thousand live births for urban areas (2007).(1) Only 31.9% of all government hospital beds are available in rural areas as compared to 68.1% for urban population. When we consider the rural-urban distribution of population in India, this difference becomes huge. Based on the current statistics provided by the Government of India, we have calculated that at a national level the current bed-population ratio for Government hospital beds for urban areas (1.1 beds/1000 population) is almost five times the ratio in rural areas (0.2 beds/1000 population).(2,3) Apart from this shortfall in infrastructure, shortfall in trained medical practitioners willing to work in rural areas is also one of the factors responsible for poor health care delivery systems in rural areas. The number of trained medical practitioners in the country is as high as 1.4 million, including 0.7 million graduate allopaths.(4) However, the rural areas are still unable to access the services of the qualified doctors. A total of 74% of the graduate doctors live in urban areas, serving only 28% of the national population, while the rural population remains largely unserved.(4) There's shortfall of 8% doctors in Primary Health Centres (PHC), 65% for specialist at Community Health centres (CHC), 55.3% for health workers (male), 12.6% for health workers (female) (2007).(5) This shortfall in human resources in rural areas is only going to increase in future, more so with corporatization and privatization of health systems. The already dwindling number of doctors in government sector and rural areas would further decrease owing to greater opportunities in private sector both in urban and peri-urban areas, and much higher remunerations.

In absence of qualified doctors, predominant providers of health care in rural areas are unqualified private practitioners, who have either no training or training in alternate system of medicine but prescribe allopathic medicines. Such providers are able to attract clientele for two reasons: firstly, non-availability of qualified doctors; and, secondly, because most of the medical conditions for which services are sought are of the common type, for which the quasi-trained practitioners can often offer some relief. However, the medical services provided by practitioners, who largely practice in a discipline in which they have no training is, in the broader context, highly damaging. For example, indiscriminate and injudicious use of antibiotics by these unqualified medical practitioners is giving rise to new mutant resistant micro-organisms.

All of us in the country, and particularly the graduate doctors' fraternity, need to reflect on why such an iniquitous and harmful mode of health service delivery exists over much of the country. Often it is argued that the financial rewards in the public health sector are too low to attract the graduate doctors to the scattered rural areas. Another reason cited is that the absence of minimal physical and social infrastructure makes it impossible for young medical graduates to serve in the rural areas, whether in public or private assignments. The qualified medical professionals willing to serve in rural areas are scarce and whatever trained doctors are available in rural areas are in government service. It is important to note here that doctors/specialists in position do not necessarily mean that doctors/specialists are physically present at their respective centres and performing their duties; in fact, absenteeism is very high. We have to be realistic and accept that trained doctors who have put in 10 years or so in training and are predominantly from urban areas are unlikely to want to go to villages. It has been suggested in various quarters that to ensure medical personnel's stay in rural areas they would have to be provided special incentives, pay hike or the basic amenities like electricity and water have to be improved to be at par with urban areas. But, overall infrastructure development cannot occur in isolation from health system development and is sure to take decades. And salaries of medical doctors cannot be disproportionately increased in relation to other service cadres.

The Task Force on Medical Education for National Rural Health Mission (NRHM) constituted by Ministry of Health and Family Welfare, Government of India was specifically asked to look into this shortfall in health personnel in rural areas and suggest various measures. It recommended increase in emoluments of doctors, re-vamping of medical education from curative to community care, compulsory rural posting and creation of new cadre of health care workers after three years of training.(6) Coercive and *ad-hoc* measures like posting medical graduates for period of two years in rural areas are unlikely to be of much use. The Ministry of Health's proposal to introduce a cadre of doctors willing to serve in rural areas after basic training of only three years met with loud protest from professional bodies like Indian Medical Association (IMA).(7) We argue that this proposed cadre of doctors, whom we would like to call Rural Health Practitioners (RHPs) is a positive step forward in the current scenario. Further, we firmly believe the existing unqualified practitioners should be roped in for training and accreditation as RHPs.

Recognizing and integrating RHP with existing health care delivery system in rural areas can be the solution for tackling this shortfall in healthcare delivery personnel. The unqualified practitioners are first point of contact for majority of rural population. In a study conducted in rural Uttar Pradesh in 1995; only 3% of medical practitioners were MBBS graduates or allopathic practitioners, while 68% of them had no training in any form of medicine.(4) The fact that they fulfil near about all criterion that one would desire from an ideal primary health care delivery worker i.e., locally available, accessible, affordable, acceptable to community, further underlines the fact that we should utilize their services. The concerns regarding their skills and malpractices are genuine, but that exist even today in absence of any regulatory mechanism. The experience with Indian laws and enforcement agencies in past and in other areas should make us realize that further tightening of laws to prevent RHPs from practicing is not a solution. Only way this concern can be addressed is by training, establishing a good supervision mechanism and developing an efficient referral system. It is considered entirely feasible to ensure that the skills available through short-course training would be fully adequate to enable RHPs to treat common conditions included in primary level of healthcare. The RHPs are already in place and may be are the only village level functionaries that are willing to serve in those areas. We just cannot wish them away or adopt an ostrich like attitude by denying their existence. By engaging them not only we can ensure quality of services provided by them but also open a new avenue of health care delivery with a focus on preventive and promotive health care at village level.

We do accept that training RHPs for health care delivery is not the ideal or optimal solution but it is not about best solution only but rather best solution within given circumstances and with given resources. As they say, “Let best not be the enemy of the good”. Any deficiency in their quality of services after training and supervision is anyway going to be much less than what it is today. Engaging them and recognizing them is the only way in which we can improve the services provided by them. As an incentive these RHPs can be provided some percentage of seats in medical colleges to become qualified doctors. Nepal has a programme to provide training and promotion opportunities for nurses and medical auxiliaries which has been hugely successful.(8)

It is also instructive to examine the experience of China, which has been far more successful in achieving basic health care for their people. It is only in the county (district) hospital that doctors trained in medical colleges, as we understand them, are found. Village clinics and Primary Health Centres are managed by Village Doctors (a new terminology replacing the old ‘Barefoot Doctors’) who are trained in preventive and curative medicine of both traditional Chinese and Allopathic schools, for periods ranging from one to three years. These skills are constantly upgraded by apprenticeship and in-service courses. A vigorous referral system operates, so that only complicated cases arrive at the county hospital. The success of barefoot doctors in China is an experience that should inspire us.

One of the potential limitations in recognizing RHPs would be medico-legal issues. We would need to amend our legal system accordingly and may be the vicarious liability for a RHP can be vested in doctors supervising them, thus, also ensuring adequate supervision. Second hurdle would be resistance by established networks/organizations of professional doctors like Indian Medical Association (IMA), Medical Council of India (MCI) etc. But, if public health professional bodies like Indian Public Health Association (IPHA) and Indian Association of Preventive and Social Medicine (IAPSM) can provide a platform for dialogue with IMA and MCI, and try to build consensus and mobilize support for this initiative, this task can very much be achieved. A third hurdle would be the apprehension that after training, the RHPs may not stay back in the rural areas. This is a pragmatic issue and combinations of possible solutions needs to be thought through, as there are no simple answers.

Indian health system is stagnated today and it requires out of box thinking, a jump start to revitalize itself. We have long been pursuing a traditional approach which has yielded limited results. If we need any further progress and improvement we have to analyze our present scenario and adapt accordingly. This rural-urban chasm needs serious deliberations at policy level and concrete steps needs to be taken to address this. There's a strong political commitment to improve rural health and NRHM was launched in 2005 with specific objective to bridge this gap. And to move from policy to implementation as far as rural health is concerned, recognizing rural health practitioners can be one of the crucial decisions.

“Primary health care is essential health care made universally accessible to individuals and acceptable to them, through their full participation and at a cost the community and country can afford”.

The rural population of India still does not get the basic quality of primary health care as stated in Alma-Ata conference attended by governments of 134 countries and many voluntary organizations in 1978 (in the former USSR). We are in 2008! Thirty years have passed since the Alma-Ata Declaration!! How long should they wait?
